# Oxaliplatin but Not Irinotecan Impairs Posthepatectomy Liver Regeneration in a Murine Model

**DOI:** 10.4061/2011/490463

**Published:** 2011-11-22

**Authors:** Perry A. Soriano, Nian Liu, Erick Castillo, Brock Foster, Avo Artinyan, Joseph Kim, Wendong Huang, Lawrence D. Wagman

**Affiliations:** ^1^Liver Tumor Program, City of Hope, 1500 E. Duarte Road, Duarte, CA 91010, USA; ^2^Center for Gene Regulation and Drug Discovery, Beckman Research Institute, City of Hope, 1500 E. Duarte Road, Duarte, CA 91010, USA; ^3^The Center for Cancer Prevention and Treatment, St. Joseph Hospital, 1000 West La Veta Avenue, Orange, CA 92868, USA

## Abstract

*Introduction*. We examined the murine hepatectomy model of liver regeneration (LR) in the setting of neoadjuvant chemotherapy. *Methods*. C57BL/6 mice were randomized to receive neoadjuvant intraperitoneal (IP) injections of a control, oxaliplatin (15 mg/kg), or irinotecan (100 mg/Kg or 250 mg/Kg) solution. Hepatectomy (70%) was performed 14 days after the final IP treatment. Animals were sacrificed at postoperative day (D) 0, 1, 2, 3, and 7. Liver remnants and serum were collected for analysis. *T*-tests for independent samples were used for statistical comparisons. *Results*. For oxaliplatin, percent LR did not differ at D1 or D2 but was significantly less at D3 (89.0% versus 70.0%, *P* = 0.048) with no difference on D7 (*P* = 0.21). Irinotecan-treated mice at both dose levels (100 mg/Kg and 250 mg/Kg) showed no significant differences in LR. BrdU incorporation was significantly decreased in oxaliplatin-treated animals (D1,2,3). *Conclusions*. Neoadjuvant oxaliplatin but not irinotecan impairs early LR in a posthepatectomy murine model which correlates with decreased DNA synthesis.

## 1. Introduction

In 2010 an estimated 142,570 people developed colorectal cancer (CRC) with an estimated 51,370 people dying of the disease [1]. Synchronous liver metastases are found in 20% of patients, and more than half of those diagnosed with CRC will go on to develop metachronous liver metastases [2, 3]. Liver only or liver-predominant disease affects 20–35% of patients, affording those with resectable lesions the possibility of long-term survival. In selected cases with R0 resection, 10-year overall survival has been reported in the literature to range from 17–25% [4, 5]. In addition to its adjuvant use in Stage 3 colon cancer and following hepatic resection, chemotherapy has the potential to convert borderline or unresectable liver disease to resectable disease by reducing the size of the tumor to an amenable dimension. Furthermore, neoadjuvant chemotherapy has been advocated as a test for aggressive tumor biology [6–8]. Timing and appropriateness of chemotherapy, however, is debated, and there are concerns regarding worse outcomes in heavily treated patients [9]. In this regard, steatohepatitis, steatosis, and sinusoidal injury have been linked to the use of irinotecan, fluoropyrimidines and oxaliplatin [10].

Animal models for the study of posthepatectomy liver regeneration are well described [11]. These models have yet to be applied to the study of commonly used agents for CRC. Given first-line use of oxaliplatin and irinotecan for stage IV CRC, these agents were chosen for investigation. We hypothesized that posthepatectomy liver regeneration is impaired by oxaliplatin and/or irinotecan administration and that this impairment can be demonstrated in a mouse model.

## 2. Materials and Methods

### 2.1. Animal Maintenance and Treatments

Eight-week-old C57BL/6 male mice, weighing between 23–25 grams, were obtained from commercial sources (Taconic Farms, Hudson, NY). The animals were housed under standard 12-hour light/12-hour dark conditions with standard feed and water ad libitum. After a minimum of 48 hours acclimation, animals were randomized to receive either oxaliplatin (15 mg/Kg), irinotecan (100 mg/kg or 250 mg/kg) or control solution (dextrose 5% water) by intraperitoneal injection. Animal tolerance of chemotherapy was closely monitored, and posthepatectomy animals were evaluated daily. Animal handling, drug administration, monitoring, and survival surgery protocols were approved by the City of Hope, Research Animal Care Committee.

### 2.2. Chemotherapy

Oxaliplatin and irinotecan were obtained through the City of Hope, Investigational Drug Services and diluted in non-chloride-containing solution (dextrose 5% water) to deliver the determined dose in an approximate volume of 100 mcL. Dose regimens were based on data from in vivo activity in previously described colon cancer tumor models in mice [12, 13]. Oxaliplatin 15 mg/Kg was administered IP × 1 dose. Irinotecan was administered at two dose levels as follows: regimen A, 100 mg/Kg, IP divided in 2 weekly doses and regimen B, 250 mg/Kg IP divided in 3 weekly doses (75 mg/Kg, 75 mg/Kg, 100 mg/Kg). Fourteen days after the last control or chemotherapy injection, a 70% hepatectomy was performed. Despite using well-established dosing schedule [12] in a dedicated vivarium with skilled personnel, 19 of 32 animals died from the initial treatment with oxaliplatin. There was no mortality in the irinotecan group. All surviving animals were included in the surgical portion of the experiment.

### 2.3. Animal Surgery

The left and median lobes were resected with preservation of the gallbladder for 70% hepatectomy. Briefly, tribromoethanol (Avertin) anesthetic was administered IP (250 mg/Kg). After sterile prep a subxiphoid transverse incision was created and the median and left liver lobes were exteriorized. The lobes were encircled with silk ligature, their vascular pedicles tied at the base and the lobes resected. Care was taken to spare the gallbladder and associated bile ducts. Closure was accomplished with autoclips. Buprenorphine was administered (0.5 mg/Kg subcutaneously) upon awakening. At postoperative days 0, 1, 2, 3, and 7, remnant right and caudate lobes were harvested, and blood was collected from the retroorbital sinus concomitant with animal sacrifice. In the oxaliplatin experimented cohort, there were 3 perioperative deaths (2 oxaliplatin treated, 1 control). There was no mortality in the irinotecan cohort.

### 2.4. Percent Liver Regeneration by Mass

Percent liver regrowth was calculated by the following formula: (Mass of regenerating liver remnant in grams) ÷ (Mass of resected liver lobes in grams)/(0.7) × 100.

### 2.5. Liver Histology and Bromodeoxyuridine (BrdU) Incorporation

In vivo BrdU staining was accomplished by intraperitoneal injection of BrdU (100 mg/Kg) 2 hours prior to sacrifice. Uniform samples of hepatic parenchyma were removed and fixed in 4% formaldehyde solution, embedded in paraffin, sectioned at 5 micrometers, and stained with hematoxylin and eosin. BrdU immunohistochemical staining was performed using a commercially available kit (Roche). The number of positively stained nuclei was counted in 3 randomly selected high-power fields per sample, one sample from at least 2 mice per time point and arm.

### 2.6. ALT Analysis

Under anesthesia prior to sacrifice, approximately 500 mcL of blood was drawn from the retro-orbital sinus and placed in serum separator tubes (Falcon). Collected serum was then analyzed for ALT after 10-fold dilution in 7% bovine serum albumin.

### 2.7. Statistical Analysis

Statistical comparisons were performed using *t*-tests for independent samples.

## 3. Results

### 3.1. Oxaliplatin

22 animals underwent 70% hepatectomy in the oxaliplatin versus control study, 9 animals in the control arm, and 13 in the oxaliplatin arm. Animal weights of the survivors were similar to those of the control group at the time of hepatectomy. There were 3 perioperative deaths; 1 in the control arm and 2 in the oxaliplatin arm which were technical in nature (pneumothorax, excessive manipulation of lobes on extraction, and hemorrhage).

**Figure 1:**
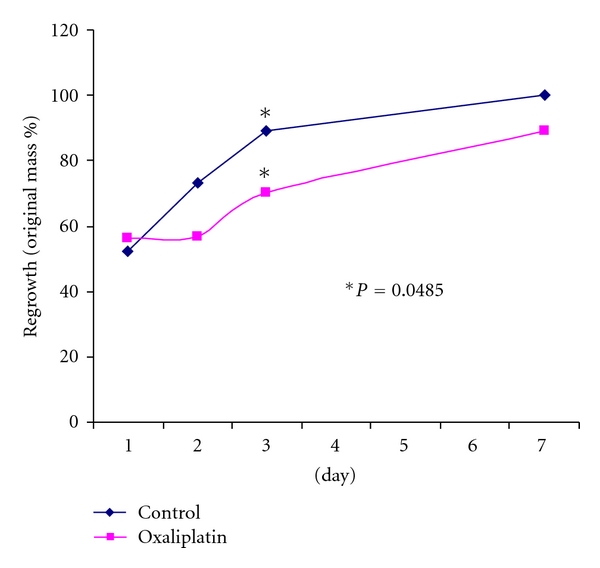
Liver regeneration by percent regrowth, oxaliplatin 15 mg/Kg versus control, differs significantly at day 3.

Percent liver regrowth (Table 1 and Figure 1) at day 1 following hepatectomy did not differ between oxaliplatin-treated and control mice (56.1% versus 52.5%, resp., *P* = 0.312). Data collected on day 2 suggests less regrowth in the oxaliplatin-treated arm (57.6% versus 73.0%, *P* = 0.154); however this was not statistically significant. Regeneration was significantly less in the treatment arm at day 3 (70.0% versus 89.0%, *P* = 0.048). By 7 days following hepatectomy, delayed LR in the oxaliplatin-treated arm was no longer found to be statically significant (89.8% versus 99.0%, *P* = 0.214).

Hepatocyte injury was assessed by measurement of ALT levels. ALT levels peaked at posthepatectomy day 1 and normalize by day 3. ALT levels in oxaliplatin-treated animals were not found to be statistically different than controls throughout the study (Figure 5). 

BrdU incorporation was used to determine if oxaliplatin impairs DNA synthesis (cellular division), thus contributing to impaired liver regrowth (Figure 3). DNA synthesis was significantly higher in the control arm at all three measured timepoints. Oxaliplatin-treated animals showed significantly less incorporation consistent with reduced DNA synthesis.

### 3.2. Irinotecan

**Figure 2:**
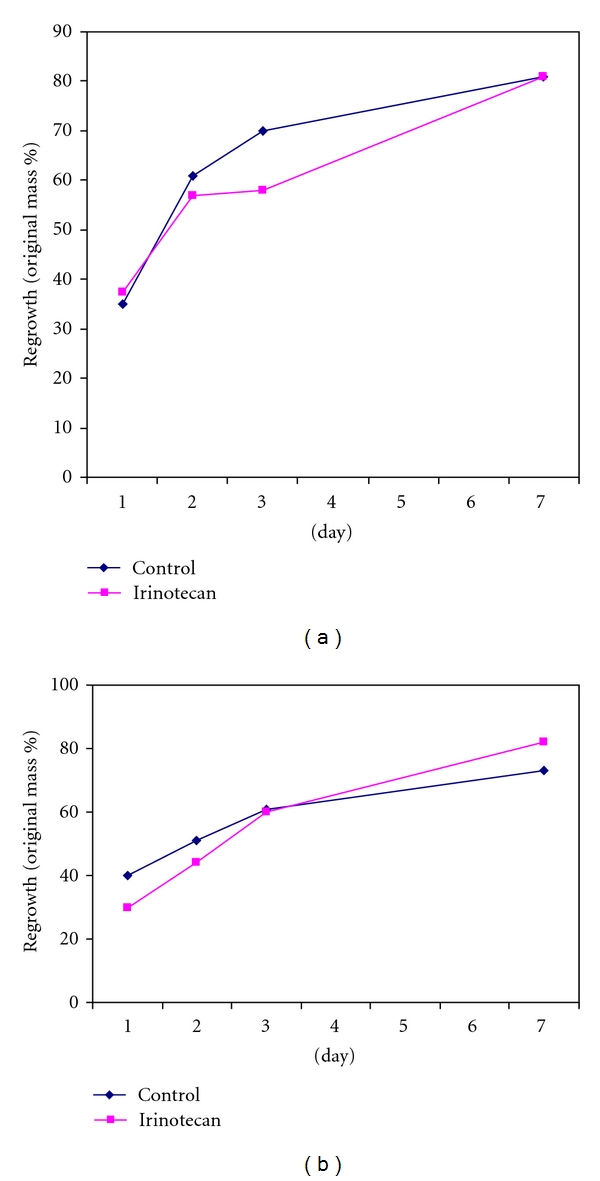
Liver regeneration by percent regrowth does not differ after irinotecan treatment at cumulative 100 mg/Kg (a) and 250 mg/Kg (b) dose levels.

**Figure 3:**
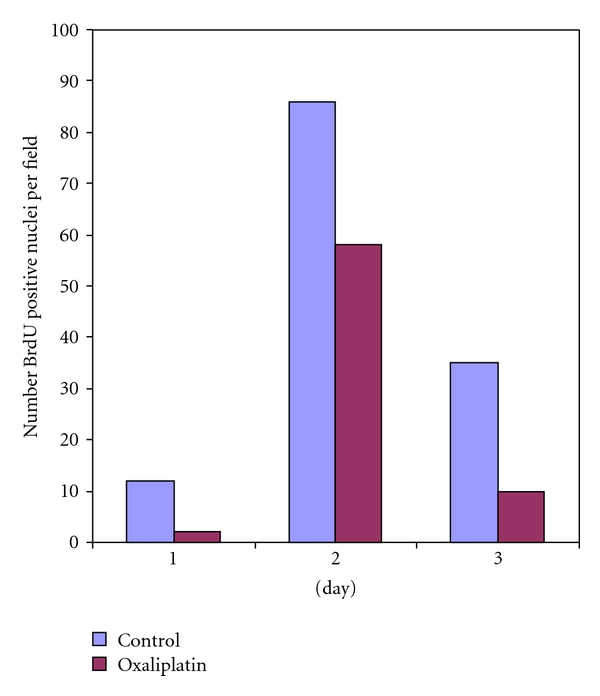
BrdU incorporation is significantly less in oxaliplatin- (15 mg/kg) treated animals at days 1, 2, and 3.

**Figure 4:**
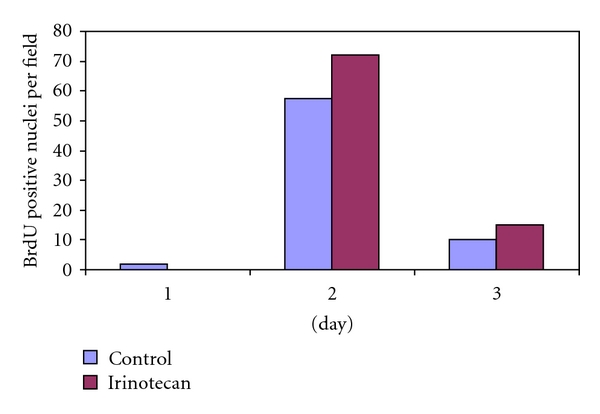
BrdU incorporation is increased in irinotecan- (100 mg/kg) treated animals.

**Figure 5:**
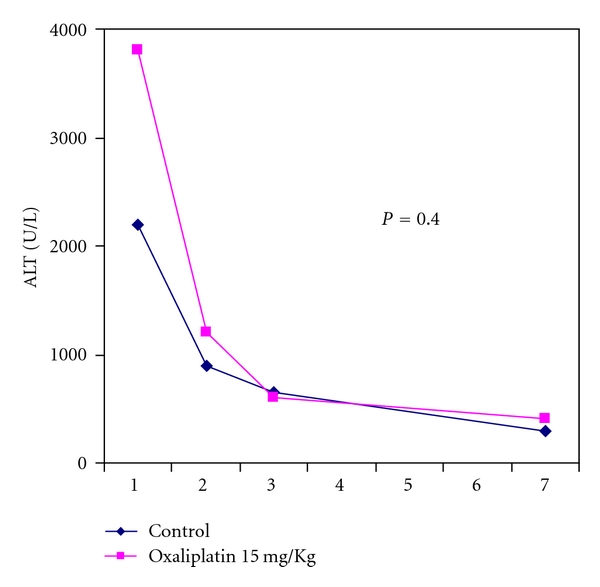
Representative plot of ALT levels showing a peak at day 1 with normalization between day 3 and 7.

In the irinotecan experiments no animals experienced chemotherapy-related mortality. Weights were similar between groups at the time of hepatectomy. Neither dose level, group A (100 mg/Kg, *N* = 15; control *N* = 8) nor group B (250 mg/Kg, *N* = 17; control *N* = 5) showed significant impairment in liver regrowth by mass compared with respective controls (Figure 2). Similar to the oxaliplatin group, irinotecan-treated animals showed peak ALT levels at day 1 with return to baseline between days 3–7 (data not shown). In contrast to the oxaliplatin results, BRDU incorporation in irinotecan-treated animals was similar or increased compared to controls (Figure 4).

### 3.3. Histology

Histologic examination of regenerating liver specimens showed no evidence of hepatic sinusoidal obstruction in oxaliplatin-or-irinotecan treated animals. Mild ballooning changes due to increased cytoplasmic water were seen in both treated and untreated groups. In the oxaliplatin arm, mild portal inflammation with necrosis near the portal triads and microvesicular steatosis were seen in two animals, one at posthepatectomy day 2 and one at day 3.

## 4. Discussion

The liver's remarkable ability to restore a functionally adequate portion of its previous volume following surgical resection is tightly regulated by mechanisms that include bile acid interactions with the FXR nuclear receptor and several other complex mechanisms [11, 14]. The mouse liver regeneration model is well described and highly reproducible in this posthepatectomy setting. The differences in regeneration are demonstrated at early timepoints, namely, days 2 and 3 after hepatectomy [11]. We chose to apply this model to the study of liver regeneration after treatment with commonly used modern chemotherapeutic agents for CRC. The oxaliplatin dose was selected based on established, species-specific doses from the research literature [12]. An unexpected toxicity (mortality) was observed in this experiment. The animal deaths affected the group sizes, but only impacted the planned experimental animal numbers (approved by the Research Animal Care Committee) that would be required to achieve definitive statistical results in one cohort.

We discovered that oxaliplatin-treated animals showed significantly reduced regrowth on the third posthepatectomy day. This finding has not been previously described in a preclinical model and may, in part, be due to the mechanism of oxaliplatin cytotoxicity. Oxaliplatin is a third-generation platinum derivative that acts at the level of DNA by forming bulky DNA adducts [15]. Most commonly, intrastrand links between guanine and adenine are formed by the platinum moiety. DNA synthesis is impaired by these adducts which in turn leads to strand breaks and subsequent apoptosis. Oxaliplatin's mechanism of action is consistent with the marked decrease in DNA synthesis demonstrated by the decrease in BrdU staining in these experiments. BrdU staining was more sensitive than percent regrowth by weight. Analysis of oxaliplatin's impact on BrdU staining were demonstrated with significant differences as early as day 1 following hepatectomy with significant impairment in DNA synthesis continuing through day 3. These data combined with the absence of direct hepatic damage, as evidenced by nonsignificant differences in ALT, suggest oxaliplatin blunts LR by inhibiting cell division in the early postoperative period. However, this effect appears to be lost by 7 days postoperatively as physiologic mechanisms to restore appropriate liver function normalize liver size by this time. Despite the consistency (growth and DNA synthetic activity) of these data, the small number of experimental animals requires they be viewed as exploratory, not definitive. The experimental model is well established, and the BrdU incorporation is a sensitive measure of DNA synthesis. The current literature contains variable conclusions on the impact of chemotherapy on liver regeneration. In part, this is due to differences in experimental modeling (e.g., number of chemotherapy injections, use of Ki-67, and single time-point analysis). The current series of experiments provides sequential time point evaluation at 0, 1, 2, 3, and 7 days in an attempt to mimic the immediate, early, and longer phases of hepatic regeneration in the human. This sequential reporting is unique in investigations of this type. This topic area remains controversial, and additional experiments with a consistent experimental model will be the most definitive way to answer the controversies and variability in results.

Irinotecan-treated animals did not show differences in liver regrowth despite treatment at previously documented pharmacologically active doses [16, 17]. BrdU incorporation assays corroborate these findings with no decrease but rather, a nonstatistically significant increase in DNA synthesis. Increased DNA synthesis with irinotecan treatment may be related to the drug's mechanism of action. Irinotecan inhibits topoisomerase I, stabilizes single-strand breaks and results in double-strand breakage though interaction at the replication fork [18]. Our results suggest that the structure and coiling of DNA is altered by irinotecan without direct effect on the cells ability to synthesize DNA and thus incorporate BrdU. Despite the somewhat counterintuitive nature of this finding, a higher proportion of cells in S-phase after irinotecan treatment has been described in animal and clinical settings [19]. 

High toxicity was seen in the animals receiving oxaliplatin. This occurred despite the use of previously reported doses [20]. In our experiments, although the toxicity was high during the administration of oxaliplatin, the surviving animals were fully recovered prior to hepatectomy with no difference in animal weight or appearance in the oxaliplatin-treated animals when compared to irinotecan-treated animals. This argues that differences in regrowth were liver specific and not a byproduct of other factors such as a weakened state or poor nutrition.

Recently, clinical studies have raised concerns regarding the significant hepatotoxicity of chemotherapeutic agents for CRC [21]. Oxaliplatin is implicated in the “blue liver” syndrome from hepatic sinusoidal obstruction, and worse posthepatectomy outcome is reported in association with chemotherapy-related steatohepatitis primarily with irinotecan [22, 23]. Histologic examination of the liver in 2 animals showed microvesicular steatosis and mild periportal inflammation. However, this was an uncommon finding. The differences seen in regeneration and DNA synthesis, therefore, likely reflect changes not yet evident on H&E, but in part detectable with special staining techniques such as BrdU immunohistochemistry. 

Clinical guidelines for hepatectomy recommend more conservative volumes of liver resection in chemotherapy-treated patients with a goal future liver remnant of 30%, rather than 20% [16, 24]. Given the adverse effects of chemotherapy on the liver, our goal was to establish an animal model to study these interactions. We have shown early impairment of regenerative ability in oxaliplatin-treated animals. These findings are corroborated by decreased DNA synthesis. These data suggest that early in the patient's postoperative course, when the risk for liver failure is higher, regenerative mechanisms may be impaired. Future studies with this model will aim at abrogating these effects.

## 5. Conclusion

The mouse 70% hepatectomy model provides a useful tool for studying the effects of chemotherapy on posthepatectomy liver regeneration. We demonstrate that oxaliplatin impairs early liver regeneration in a posthepatectomy model and that this reduced regrowth correlates with decreased DNA synthesis. Conversely, irinotecan did not impair regeneration or DNA synthesis.

## Figures and Tables

**Table 1:** Liver regeneration after oxaliplatin treatment, percent regrowth by mass, days 1, 2, 3, and 7.

Day	Regrowth control	Regrowth oxaliplatin	Independent *T*-test
Day 1	56.1% (*N* = 2)	52.5% (*N* = 3)	*P* = 0.312
Day 2	73.0% (*N* = 2)	57.6% (*N* = 3)	*P* = 0.154
Day 3	89.0% (*N* = 2)	70.0% (*N* = 3)	*P* = 0.048
Day 7	99.0% (*N* = 2)	89.8% (*N* = 2)	*P* = 0.214

*N*, number of animals per group.
